# Association of critical thinking disposition with personality traits and differentiation of self in medical undergraduates, a multicenter cross-sectional study in China

**DOI:** 10.3389/fmed.2025.1561786

**Published:** 2025-06-17

**Authors:** Zixuan Zeng, Xiaohan Wang, Hengxing Sun, Jessica Thai, Yafen Gan, Enxiu Li, Lei Huang

**Affiliations:** ^1^Department of Psychiatry, Tongji Hospital, School of Medicine, Tongji University, Shanghai, China; ^2^School of Medicine, Tongji University, Shanghai, China; ^3^James A. Haley Veterans’ Hospital, Tampa, FL, United States; ^4^Gannan Normal University, Ganzhou, Jiangxi, China; ^5^Medical College of Soochow University, Suzhou, Jiangsu, China; ^6^Department of Medical Education, Tongji Hospital, School of Medicine, Tongji University, Shanghai, China

**Keywords:** critical thinking, differentiation of self, medical students, personality, undergraduates

## Abstract

**Background:**

Critical thinking is one of the seven essential competencies of the Global Minimum Essential Requirements in Medical Education. It is essential to cultivate medical students’ critical thinking as it influences their clinical decision-making. The undergraduate years represent a critical period for medical students in terms of personality development and self-differentiation, which are essential foundations for shaping critical thinking. Therefore, this multicenter cross-sectional study aimed to explore the relationships of critical thinking disposition with personality traits and differentiation of self in medical undergraduates.

**Methods:**

A total of 1,338 medical students from three institutions in China were selected for this study using a stratified cluster sampling method. The Critical Thinking Disposition Inventory-Chinese Version (CTDI-CV), Eysenck Personality Questionnaire (EPQ) and Differentiation of Self Inventory-Revised (DSI-R) were applied to assess medical students’ critical thinking disposition, personality traits and differentiation of self. Multiple linear regression analysis was conducted to test the relationships of critical thinking disposition with personality traits and differentiation of self. Binary logistic regression model was established for sensitivity analysis.

**Results:**

Linear regression analysis showed that psychoticism and neuroticism could negatively influence critical thinking disposition [β, 95% confidence interval (CI) = −0.363 (−0.411, −0.316); −0.129 (−0.189, −0.070)]. Conversely, extraversion and differentiation of self could positively influence critical thinking disposition [β, 95% CI = 0.145 (0.096, 0.194); 0.279 (0.224, 0.334)]. The results of the binary logistic regression were consistent with those of the linear regression model.

**Discussion:**

This study suggested the potential need for tailored critical thinking development strategies for medical students with different personality traits and degrees of differentiation of self.

## Introduction

Critical thinking (CT) is defined as an active mental process of purposeful and self-regulatory judgment, which entails interpretation, evaluation, analysis, and inference (1). Listed as one of the seven essential competencies of the Global Minimum Essential Requirements in Medical Education (GMER) by the Institute for International Medical Education (IIME), CT is essential for professional competence in medical students (2, 3).

According to Facione’s self-regulation theory, physicians’ CT includes critical thinking skills (CTS) and critical thinking disposition (CTD) (4, 5). CTD, considered a relatively stable personality-related trait, refers to one’s intrinsic motivation to engage in thoughtful and reflective problem-solving (5, 6). It encompasses seven key dimensions: truth-seeking, open-mindedness, analyticity, systematicity, self-confidence, inquisitiveness, and maturity. Prior studies have shown that stronger CTD is associated with higher self-esteem, academic performance, innovation, and clinical competence in physicians (7–14). CTD begins to form during undergraduate training, a critical stage in the development of clinical reasoning and decision-making abilities. Therefore, exploring the factors influencing CTD in medical undergraduates is vital for developing effective educational interventions. While previous research has focused on educational factors such as teaching methods and learning styles (15–18), recent studies highlight the importance of individual psychological characteristics (19–22).

Among individual traits, personality factors have attracted increasing attention. Eysenck’s Personality Theory posits that personality traits can be categorized into three primary dimensions: extroversion-introversion, neuroticism-stability, and psychoticism (23). These personality traits influence learning behaviors and cognitive styles. For instance, extroversion is often linked with curiosity and openness to new ideas, whereas neuroticism and psychoticism are associated with emotional instability and rigidity (23). Fu and her colleagues found negative associations between neuroticism and psychoticism and CTS among Chinese nurses, while extraversion demonstrated a positive association (24). Similarly, other studies have indicated that medical students with personality traits such as extroversion, stability, flexibility, and agreeableness were more likely to become self-directed and independent thinkers (25–27). Despite this, limited research has explored the relationship between personality traits and CTD in Chinese medical students.

In addition to personality, differentiation of self (DS), a concept from Bowen’s Family Systems Theory, may also influence CTD. DS is defined as individuals’ ability to balance emotional and intellectual functioning, as well as intimacy and autonomy, within relationships (28). Bowen held that DS was one of the most important qualities for individual maturity and mental health, and it was a necessary growth goal for individuals and the family (28, 29). Those with higher DS tend to think objectively under stress and are less swayed by others’ opinions—traits consistent with CTD (25, 30) Knauth and Shams found that the level of DS may influence the ability of social problem solving and the styles of decision-making in adolescents (31, 32). However, there has been limited research exploring the relationship between DS and CTD.

Personality traits and DS reflect two complementary aspects of individual psychological functioning—one rooted in temperament and cognition, the other in emotional regulation and interpersonal autonomy. Investigating their combined effects on CTD may provide a more comprehensive understanding of how individual factors shape critical thinking. In Chinese culture specifically, where collectivism and social harmony are emphasized, both personality development and DS may follow different patterns compared to Western populations (33, 34). These cultural influences may, in turn, affect the development of CTD among Chinese medical students. Therefore, exploring and understanding the relationship of CTD with personality traits and DS is crucial for designing targeted interventions to foster CTD among Chinese medical students.

To our knowledge, this is the first study to investigate the influence of personality traits and DS on CTD in medical undergraduates in China. This study has two hypotheses. First, medical undergraduates with higher levels of extroversion and lower levels of psychoticism and neuroticism traits are more likely to exhibit positive CTD. Second, the level of DS may positively influence CTD.

## Materials and methods

### Research design

This was a multi-center cross-sectional study conducted at three universities in China: Tongji University School of Medicine (Shanghai), Medical College of Soochow University (Jiangsu province), and Gannan Medical University (Jiangxi province). They reflect different institutional tiers within China’s medical education system. This selection ensures that our sample captures a broad spectrum of medical students from different educational backgrounds, which enhances the generalizability and representativeness of our findings. The study was approved by the Tongji Hospital of Tongji University Institutional Review Board (Registration Number K-2014-020); Medical College of Soochow University Institutional Review Board (Registration Number SUDA20210122H02) and Gannan Medical University Institutional Review Board (Registration Number 2014468). All respondents signed an informed consent form before participating in the investigation. This study was carried out and reported following STROBE statement (Supplementary Table 1).

### Participants

In China, medical students typically complete a 5-year undergraduate education program to earn a Bachelor’s degree. The study used a stratified cluster sampling method. Specifically, we aimed to sample approximately 80 to 100 students per grade (from grade 1 to grade 5) from each school. The eligible criteria were (1) medical undergraduates from the above three universities; (2) a major in clinical medicine; (3) willingness to participate in this research study.

### Measurements

#### Critical Thinking Disposition Inventory-Chinese Version (CTDI-CV)

The Chinese Critical Thinking Disposition Inventory (CTDI-CV) was used to measure medical students’ critical thinking disposition. It was translated and modified by Peng (35) according to Facione’s (36) California Critical Thinking Dispositions Inventory (CCTDI). The CTDI-CV is a standardized 70-item multiple-choice test that examines seven dimensions of CTD including “Truth seeking,” “Open-mindedness,” “Self-confidence,” “Inquisitiveness,” “Cognitive maturity,” “Analyticity,” and “Systematicity.” Each dimension contained 10 items, and each item was rated on a 6-point Likert scale. Varying from 70 to 420, a total score over 280 suggests a positive attitude toward CTD (35). The CTDI-CV demonstrated good reliability and validity, with an overall Cronbach’s α of 0.90, sub-scale Cronbach’s α ranging from 0.65 to 0.81, and a Content Validity Index (CVI) of 0.89 (35).

#### Eysenck Personality Questionnaire (EPQ)

The Eysenck Personality Questionnaire was compiled by a British clinical psychologist, Eysenck (23). The Chinese version of the questionnaire was presided by Gong (37). The number of items in the revised version was changed from 90 to 88 and includes 4 sub-scales: “Psychoticism” (EPQ-P), “Extraversion” (EPQ-E), “Neuroticism” (EPQ-N), and “Lying” (EPQ-L). In this questionnaire, participants were asked to answer yes (1) or no (0) to each item. The EPQ has been widely used among Chinese medical students (38). Since the EPQ-L was used to assess the reliability of participants’ responses, the results of this dimension were excluded from the analysis in our study. The Cronbach’s α of E, N, P were 0.83, 0.88 and 0.84, respectively. Test-retest reliability showed significant correlations (P: *r* = 0.60–0.65; E: *r* = 0.58–0.86; N: *r* = 0.64–0.73) (37).

#### Differentiation of Self Inventory-Revised (DSI-R)

The Differentiation of Self Scale Inventory-Revised, developed by Wu and Wang (39), was used to measure the differentiation of self in both intrapersonal and interpersonal dimensions. The DSI-R was translated and modified according to the Differentiation of Self Inventory (DSI) (40). This 27-item self-report questionnaire consisted of 4 sub-scales: “Emotional Reactivity (ER),” “I-Position (IP),” “Emotional Cutoff (EC),” and “Fusion with Others (FO).” It has been widely used among Chinese students (41). Each item was rated with a 6-point Likert scale ranging from 1 to 6. Higher total scores indicated better self-differentiation. The scale demonstrated good reliability with a Cronbach’s α coefficient of 0.86, and sub-scale Cronbach’s α values ranging from 0.62 to 0.77. The test-retest reliability for the total scale was 0.77 (41).

#### Covariates

Covariates include demographics that may have an influence on critical thinking disposition. We collected information on age, sex (male or female), school (Gannan Medical University, Medical College of Soochow University, Tongji university School of Medicine), year of study (from first to fifth), only child status in family (yes or no), whether students voluntarily majored in medicine (yes or no), and willingness to be a doctor in the future (yes or no). Age was divided into two categories (< 22 or ≥ 22) based on the average response value.

### Statistical analysis

Descriptive statistics were computed using means and standard deviations (SD) as continuous variables, and frequencies and percentages as categorical variables. To compare CTD score differences between groups, we used two independent sample *t*-test or one-way ANOVA methods. Pearson’s correlation was used to assess the degree of association among personality traits, DS, and CTD. Multiple linear regression was used to analyze the relationships between CTD, personality traits, and DS. While our sample includes students from three different schools, the primary focus of this study was to examine the overall relationships between these variables, rather than school-level differences. Therefore, treating the sample as a whole and applying multiple linear regression was a appropriate method to maintain statistical power and clarity in the analysis. Complementarily, binary logistic regression served as a sensitivity analysis by categorizing CTD scores into positive and negative attitude groups using a cutoff of 280 (35), offering predictive probabilities for binary outcomes and validating findings through alternative classification. Both regression models incorporated covariates. When examining the relationship between DS and CTD, demographics and personality traits were controlled as covariates. When investigating the relationship between personality and CTD, demographics and DS were treated as covariates. All statistical procedures were conducted using SPSS version 25.0. Two-tailed *p* < 0.05 was considered statistically significant.

## Results

### Participant characteristics

A total of 1,338 medical undergraduates were recruited for this study. The average age of the participants was 22.08 ± 1.74 years. Nearly half of the participants were male (49.8%) and the only child (50.4%) in family. 295 (22.0%) were first year students, 252 (18.8%) were in their second year, 300 (22.4%) were in their third year, 289 (21.6%) were in their fourth year, and the rest were in their fifth year. More than half (56.4%) of the students chose to study medicine voluntarily, and most of them (91.1%) were willing to be a doctor in the future. Other details are shown in Tables 1, 2.

**TABLE 1:** Comparison of the CTDI-CV total scores according to demographics.

	Category	Overall (*n* = 1,338)			
		***n* (%)**	**Mean ± SD**	***t*/*F***	***p***
**Age**					
	< 22	514 (38.4)	290.73 ± 28.65	3.46	0.001
	≥ 22	824 (61.6)	284.99 ± 30.10		
**Sex**					
	Male	667 (49.8)	285.8 ± 30.86	−1.67	0.096
	Female	671 (50.2)	288.5 ± 28.40		
**School**					
	Gannan Medical University	482 (36.0)	285.5 ± 29.72	4.81	0.008
	Medical College of Soochow University	388 (29.0)	285.2 ± 27.36		
	Tongji University School of Medicine	468 (35.0)	290.6 ± 31.19		
**Year of study**					
	First year	295 (22.0)	289.1 ± 27.00	6.76	< 0.001
	Second year	252 (18.8)	293.4 ± 29.37		
	Third year	300 (22.4)	285.8 ± 28.02		
	Fourth year	289 (21.6)	286.8 ± 32.10		
	Fifth year	202 (15.1)	279.5 ± 30.86		
**Only child**					
	Yes	674 (50.4)	287.7 ± 31.07	0.60	0.550
	No	664 (49.6)	286.7 ± 28.20		
**Voluntarily majored in medicine**					
	Yes	754 (56.4)	290.0 ± 29.36	3.94	< 0.001
	No	584 (43.6)	283.6 ± 29.71		
**Willingness to be a doctor**					
	Yes	1,219 (91.1)	287.8 ± 29.26	2.17	0.030
	No	119 (8.9)	281.6 ± 33.24		

CTDI-CV, Critical Thinking Disposition Inventory-Chinese Version; SD, standard deviation. Group differences were analyzed by two independent sample *t*-tests (dichotomous variables) or one-way ANOVA methods (polytomous variables).

**TABLE 2:** Comparison of positive and negative attitude toward CTD according to demographics.

	Category	Positive CTD (*n* = 772)	Negative CTD (*n* = 556)	χ^2^	*p*
		*N*	*N*		
**Age**					
	< 22	329	185	13.61	< 0.001
	≥ 22	443	381		
**Sex**					
	Male	366	301	4.35	0.037
	Female	265	406		
**School**					
	Gannan Medical University	270	212	3.45	0.179
	Medical College of Soochow University	216	172		
	Tongji University School of Medicine	286	182		
**Year of study**					
	First year	187	108	20.95	< 0.001
	Second year	161	91		
	Third year	173	127		
	Fourth year	159	130		
	Fifth year	92	110		
**Only child**					
	Yes	390	284	0.02	0.902
	No	382	282		
**Voluntarily majored in medicine**					
	Yes	477	277	21.92	< 0.001
	No	295	289		
**Willingness to be a doctor**					
	Yes	715	504	5.14	0.023
	No	57	62		

CTD, critical thinking disposition.

The mean ± SD scores of and EPQ.P, EPQ.E, EPQ.N, DSI-R, and CTDI-CV were 5.2 ± 3.24, 11.9 ± 4.45, 12.4 ± 5.14, 101.0 ± 14.26, and 287.2 ± 29.67, respectively. 772 (57.7%) participants had a CTDI-CV total score over 280, indicating a positive attitude toward CTD. In contrast, 556 (42.3%) had a negative attitude to CTD.

### Differences between demographics and CTDI-CV total scores and attitude

Tables 1, 2 showed that participants under the age of 22, those who voluntarily chose to major in medicine, and those who expressed willingness to become a doctor in the future tended to have higher CTDI-CV total scores and a more positive attitude toward CTD (all *p* < 0.05). Although a significant sex difference was observed in attitudes toward CTD (*p* = 0.037), no significant difference was found in CTDI-CV total scores between males and females (*p* = 0.096). Students from different schools (*p* = 0.008) and years of study (*p* < 0.001) had different CTDI-CV total scores. However, there were no differences in CTDI-CV total scores or attitudes between students who were only children and those who were not (*p* > 0.05).

### Relationship between personality traits, DS and CTD

**FIGURE 1:**
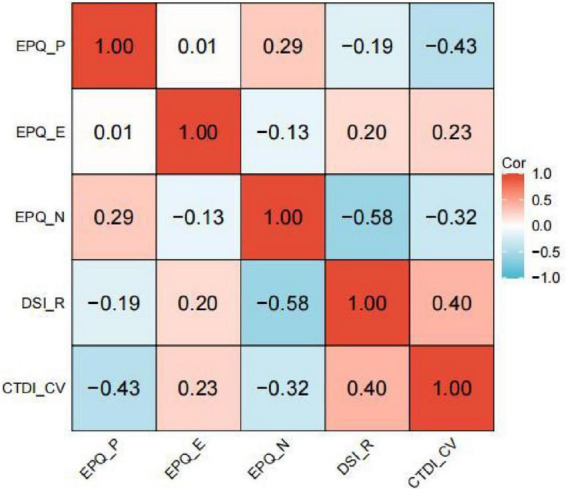
Heatmap of Pearson correlations among EPQ subscales, DSI-R and CTDI-CV total scores. EPQ, Eysenck Personality Questionnaire; P, psychoticism; E, extraversion; N, neuroticism; DSI-R, Differentiation of Self Inventory-Revised; CTDI-CV, Critical Thinking Disposition Inventory-Chinese Version; all *p* < 0.05.

The Pearson’s correlation coefficients (Figure 1) demonstrated that CTDI-CV scores were significantly associated with EPQ.P, EPQ.E, EPQ.N and DSI-R scores (*r* = −0.426, 0.227, −0.319, 0.404, all *p* < 0.001). The results of Pearson correlation between personality traits, differentiation of self, critical thinking disposition and their sub-dimensions were displayed at Supplementary Table 2.

The multiple linear regression model showed that psychoticism and neuroticism could negatively influence with CTD [β = −0.426, 95% CI (−0.474, −0.377), *p* < 0.001; β = −0.319, 95% CI (−0.370, −0.268), *p* < 0.001]. Conversely, extraversion and DS could positively influence CTD [β = 0.227, 95% CI (0.175, 0.280), *p* < 0.001; β = 0.404, 95% CI (0.354, 0.453), *p* < 0.001]. After adjusting for the covariates, respectively, the relationships between psychoticism, neuroticism, extraversion, and DS remained nearly unchanged [β = −0.363, 95% CI (−0.411, −0.316), *p* < 0.001; β = −0.129, 95% CI (−0.189, −0.070), *p* < 0.001; β = 0.145, 95% CI (0.096, 0.194), *p* < 0.001; β = 0.279, 95% CI (0.224, 0.334), *p* < 0.001]. This suggested that psychotic and neurotic personalities may lead to weaker CTD, and extraverted personality traits may foster stronger CTD.

When we divided CTD into positive and negative attitudes and established the logistic model, the results suggested that higher scores of psychoticism and neuroticism could significantly negatively influence CTD [OR = 0.786, 95% CI (0.756, 0.818), *p* < 0.001; OR = 0.903, 95% CI (0.883, 0.924), *p* < 0.001] while higher scores of extraversion and DS could significantly positively influence CTD [OR = 1.089, 95% CI (1.062, 1.117), *p* < 0.001; OR = 1.053, 95% CI (1.043, 1.063), *p* < 0.001]. After adjusting for the covariates, respectively, the relationships remained unchanged. Overall, the results of the binary logistic regression were consistent with those of the linear regression model, indicating the robustness of the findings in this study. Details are displayed further in Table 3.

**TABLE 3:** Multiple linear regression and binary logistic regression models of CTD with personality traits and differentiation of self.

	Multiple linear regression		Binary logistic regression	
	Non-adjusted β (95% CI)	Adjusted β (95% CI)	Non-adjusted OR (95% CI)	Adjusted OR (95% CI)
EPQ.P	−0.426*** (−0.474, −0.377)	−0.363*** (−0.411, −0.316)^a^	0.786*** (0.756, 0.818)	0.803*** (0.769, 0.838)^a^
EPQ.E	0.227*** (0.175, 0.280)	0.145*** (0.096, 0.194)^a^	1.089*** (1.062, 1.117)	1.065*** (1.037, 1.095)^a^
EPQ.N	−0.319*** (−0.370, −0.268)	−0.129*** (−0.189, −0.070)^a^	0.903*** (0.883, 0.924)	0.959** (0.932, 0.987)^a^
DSI-R	0.404*** (0.354, 0.453)	0.279*** (0.224, 0.334)^b^	1.053*** (1.043, 1.063)	1.042*** (1.030, 1.054)^b^

The dependent variable of multiple linear models was the total score of CTDI-CV. The dependent variable of binary logistic regression was positive CTD (using negative CTD as a reference).

*^a^*Adjusted for covariates, including age, sex, school, year of study, voluntarily majored in medicine, willingness to be a doctor, and DSI-R total score.

*^b^*Adjusted for covariates, including age, sex, school, year of study, voluntarily majored in medicine, willingness to be a doctor, and total scores of EPQ.P, EPQ.E and EPQ.N. EPQ, Eysenck Personality Questionnaire; P, psychoticism; E, extraversion; N, neuroticism; DSI-R, Differentiation of Self Inventory-Revised; CTDI-CV, Critical Thinking Disposition Inventory-Chinese Version; OR, odds ratio; CI, confidence interval.

**p < 0.01;

***p < 0.001.

## Discussion

The undergraduate years represent a critical period for medical students in terms of personality development and self-differentiation, which are essential foundations for shaping CTD. The cultivation of CT is crucial for their future careers. This study found that psychotic and neurotic personality traits were negatively associated with CTD, while extraversion was positively associated with CTD. These findings suggest the potential need for tailored critical thinking development programs for medical students with different personality traits. Furthermore, to the best of our knowledge, this is one of the earliest studies to explore the relationship between DS and CTD in medical students. The results indicate that individuals with higher DS scores tend to have higher CTD scores, suggesting that promoting DS in medical students may help foster their CTD.

In this study, medical students with higher levels of psychoticism tended to have lower levels of CTD, which is congruent with Zeng’s (42) findings. This aligns with the Eysenck’s Personality theoretical framework suggesting that psychoticism was associated with traits such as isolation, paranoia, and hostility, all of which could hinder the development of CTD (23). As Eysenck defined, psychoticism is “a dispositional variable or trait predisposing people to functional psychotic disorders of all types.” Therefore, individuals with high levels of psychoticism may struggle with the objectivity, open-mindedness, and the systematic approach required for CT, as their emotional reactivity and lack of empathy may interfere with rational decision-making and collaborative problem-solving (43, 44). Additionally, medical students with elevated psychoticism may display resistance to feedback and a reduced ability to engage in self-reflection, which are essential for cultivating CTD (45). These results highlight the importance of early identification and targeted interventions for medical students with high psychoticism levels. Tailored educational strategies, such as reflective practice exercises could help mitigate the negative effects of psychoticism on CTD (46).

The negative predictive relationship between neuroticism and CTD observed in this study is consistent with findings by Buzduga (47), indicating that higher levels of neuroticism are linked to lower CTD. Medical students with high degrees of neuroticism tend to display emotional instability, excessive worry, and difficulties managing stress, which may impair cognitive flexibility and objectivity (48). Meanwhile, higher levels of neuroticism have also been shown to correlate with poorer mental health (49). Liu and colleagues suggested that mental health was associated with both CTD and CTS (50). This indicates that neuroticism may indirectly affect CTD by influencing mental health, underscoring the potential value of integrating mental health support into medical education to foster CT development. Programs focusing on stress management such as mindfulness-based intervention (MBI) may help enhance the ability to approach problems with greater critical thinking (51).

This study found that extraversion could positively influence CTD, which supports previous research findings that extraverted medical students may have more positive CTD than introverted students (52). Extraverted students tend to be more sociable, adaptable, and open to unknown knowledge and new experiences. These traits may enhance their curiosity and willingness to engage with diverse perspectives, enabling them to actively analyze and process new information (53). Consequently, these qualities may promote the development of positive CTD.

The results showed that DS could positively influence CTD among medical undergraduates. Three potential explanations may account for this finding. First, DS is closely linked to interpersonal relationships and communication patterns (54). Highly differentiated individuals are better at balancing personal boundaries and respecting others’ opinions. This promotes diverse perspectives, helping individuals recognize unconscious biases and adopt more open-minded and tolerant outlooks, which are key traits of effective critical thinkers (55). Secondly, highly differentiated individuals tend to exhibit lower stress responses and more positive coping styles (56). Coping styles, defined as strategies used to address unexpected events, have been reported to be positively associated with CTD (57). Individuals with positive coping styles actively seek out and gather information, while taking personal responsibility, gaining self-confidence through successful experiences. This aligns with the “Self-confidence” dimension of CTD, playing a pivotal role in its development. Thirdly, highly differentiated individuals may have higher resilience and stronger emotional regulation abilities (58). Highly differentiated individuals are more resilient, adapt dynamically to challenges, and maintain composure, enabling them to think independently and exercise sound judgment in complex situations (59, 60). These traits are critical for the development of CTD. Medical education should therefore prioritize fostering both emotional and intellectual maturity to enhance students’ abilities to think critically.

This study advances the theoretical understanding of CTD development by integrating personality traits and self-differentiation within the unique sociocultural context of Chinese medical education. Bowen’s Family Systems Theory posits that DS is a critical marker of emotional and cognitive maturity, enabling individuals to balance autonomy and intimacy while maintaining rational judgment (61). Our findings extend this framework by demonstrating that DS operates as a robust predictor of CTD in Chinese medical students, even after controlling for personality traits. This underscores the importance of fostering emotional resilience and boundary-setting skills in collectivist cultures, where hierarchical norms and group harmony are prioritized, potentially stifling independent thought (62). By highlighting DS as a modifiable target, our work suggests that interventions promoting intrapersonal and interpersonal maturity, such as Balint groups or reflective practice training, could counterbalance cultural pressures that inhibit critical thinking.

The Balint Group, with its focus on offering a confidential and supportive environment for exploring professional beliefs, values, and emotions, may be an effective way to promote DS and CTD in medical students (63, 64). By encouraging personal reflection and addressing emotional and cognitive challenges, Balint Groups may enhance critical thinking and promote a more balanced approach to medical practice. This structured dialog enhances self-awareness, challenges unconscious biases, promotes cognitive skills, and ultimately fosters the development of DS and CTD. Research on Balint groups have observed that participants’ empathy improved, defensive communication decreased, and clinical reasoning abilities increased, which is consistent with current research on CTD (65). Thus, incorporating Balint Groups into medical education could be a valuable strategy for nurturing both DS and CTD in future physicians. However, its implementation is still rare in Chinese medical undergraduates.

Cross-culturally, our results diverge from Western studies, where individualism and autonomy are more culturally sanctioned (66). For instance, while extraversion consistently predicts CTD across cultures, the strength of the DS-CTD association in our sample may reflect compensatory mechanisms in collectivist settings (67). Chinese medical students, socialized to prioritize group consensus, may rely more heavily on DS to navigate conflicting professional and familial expectations. Similarly, the pronounced negative effects of psychoticism and neuroticism on CTD align with Eysenck’s model but may be exacerbated by cultural stigma around mental health, which could further impair critical engagement.

This study has several highlights. Firstly, this study represents the first attempt to our knowledge to explore the relationships among personality traits and differentiation of self and critical thinking dispositions simultaneously among medical students. Secondly, our sample was relatively large, and findings of this study proved stable through sensitive analyses. This study also had some limitations. Firstly, this was a cross-sectional survey, so no causality could be established between variables. Secondly, the use of self-reported assessments could reduce the data’s validity. Future studies could consider adopting a longitudinal design and exploring the potential mechanisms of interactions among personality traits, differentiation of self and critical thinking.

## Conclusion

This study highlights the significant role of personality traits and differentiation of self in shaping critical thinking among Chinese medical students. Our findings reveal that psychoticism and neuroticism negatively impact CTD, while extraversion and higher DS levels promote its development. These results underscore the importance of considering personality characteristics and emotional maturity in the design of educational strategies aimed at fostering critical thinking in medical education.

## Data Availability

The raw data supporting the conclusions of this article will be made available by the authors, without undue reservation.
