# Supplementary material for: Association of critical thinking disposition with personality traits and differentiation of self in medical undergraduates, a multicenter cross-sectional study in China
Source: Front Med (Lausanne). 2025 Jun 17;12:1561786. doi: 10.3389/fmed.2025.1561786 (PMC12209401; doi:10.3389/fmed.2025.1561786)
Supplement: Supplementary file 1 [file Data_Sheet_1.pdf]

Supplementary Material

Association of critical thinking disposition with personality traits and differentiation of self in medical undergraduates, a multicenter cross-sectional study in China

Zixuan Zeng[1], Xiaohan Wang[2], Hengxing Sun[2],Jessica Thai[3], Yafen Gan[4], Enxiu Li[5] and Lei Huang[1,6]*

*** Correspondence:** Lei Huang: huanglei@tongji.edu.cn

1 Supplementary Tables

Supplementary Table S1: STROBE Statement—checklist of items that should be included in reports of observational studies

STROBE Statement—checklist of items that should be included in reports of observational studies

	Item No.	Recommendation	Page No.
**Title and abstract**	1	(*a*) Indicate the study's design with a commonly used term in the title or the abstract	1
		(*b*) Provide in the abstract an informative and balanced summary of what was done and what was found	1
**Introduction**			
Background/rationale	2	Explain the scientific background and rationale for the investigation being reported	2
Objectives	3	State specific objectives, including any prespecified hypotheses	2
**Methods**			
Study design	4	Present key elements of study design early in the paper	3
Setting	5	Describe the setting, locations, and relevant dates, including periods of recruitment, exposure, follow-up, and data collection	3
Participants	6	(*a*) *Cohort study*—Give the eligibility criteria, and the sources and methods of selection of participants. Describe methods of follow-up<br>*Case-control study*—Give the eligibility criteria, and the sources and methods of case ascertainment and control selection. Give the rationale for the choice of cases and controls<br>*Cross-sectional study*—Give the eligibility criteria, and the sources and methods of selection of participants	3
		(*b*) *Cohort study*—For matched studies, give matching criteria and number of exposed and unexposed<br>*Case-control study*—For matched studies, give matching criteria and the number of controls per case	Not applicable
Variables	7	Clearly define all outcomes, exposures, predictors, potential confounders, and effect modifiers. Give diagnostic criteria, if applicable	3-4
Data sources/ measurement	8*	For each variable of interest, give sources of data and details of methods of assessment (measurement). Describe comparability of assessment methods if there is more than one group	3-4
Bias	9	Describe any efforts to address potential sources of bias	4
Study size	10	Explain how the study size was arrived at	none

Continued on next page

Quantitative variables	11	Explain how quantitative variables were handled in the analyses. If applicable, describe which groupings were chosen and why	4
Statistical methods	12	(*a*) Describe all statistical methods, including those used to control for confounding	4
		(*b*) Describe any methods used to examine subgroups and interactions	4
		(*c*) Explain how missing data were addressed	Not applicable
		(*d*) *Cohort study*—If applicable, explain how loss to follow-up was addressed<br>*Case-control study*—If applicable, explain how matching of cases and controls was addressed<br>*Cross-sectional study*—If applicable, describe analytical methods taking account of sampling strategy	Not applicable
		(*e*) Describe any sensitivity analyses	4
Participants	13*	(a) Report numbers of individuals at each stage of study—eg numbers potentially eligible, examined for eligibility, confirmed eligible, included in the study, completing follow-up, and analysed	4
		(b) Give reasons for non-participation at each stage	Not applicable
		(c) Consider use of a flow diagram	Not applicable
Descriptive data	14*	(a) Give characteristics of study participants (eg demographic, clinical, social) and information on exposures and potential confounders	4
		(b) Indicate number of participants with missing data for each variable of interest	Not applicable
		(c) *Cohort study*—Summarise follow-up time (eg, average and total amount)	Not applicable
Outcome data	15*	*Cohort study*—Report numbers of outcome events or summary measures over time	Not applicable
		Case-control study—Report numbers in each exposure category, or summary measures of exposure	Not applicable
		Cross-sectional study—Report numbers of outcome events or summary measures	4
Main results	16	(*a*) Give unadjusted estimates and, if applicable, confounder-adjusted estimates and their precision (eg, 95% confidence interval). Make clear which confounders were adjusted for and why they were included	4-7
		(*b*) Report category boundaries when continuous variables were categorized	4
		(*c*) If relevant, consider translating estimates of relative risk into absolute risk for a meaningful time period	Not applicable

Continued on next page

Other analyses	17	Report other analyses done—eg analyses of subgroups and interactions, and sensitivity analyses	4-7
Key results	18	Summarise key results with reference to study objectives	7
Limitations	19	Discuss limitations of the study, taking into account sources of potential bias or imprecision. Discuss both direction and magnitude of any potential bias	8
Interpretation	20	Give a cautious overall interpretation of results considering objectives, limitations, multiplicity of analyses, results from similar studies, and other relevant evidence	7-8
Generalisability	21	Discuss the generalisability (external validity) of the study results	8
**Other information**			
Funding	22	Give the source of funding and the role of the funders for the present study and, if applicable, for the original study on which the present article is based	8

Supplementary Table 2. Pearson Correlation between personality traits, differentiation of self, critical thinking disposition and their sub-dimensions

Variables	Truth seeking	Open-mindedness	Analyticity	Systematicity	Self-confidence	Inquisitiveness	Cognitive maturity	CTDI-CV total score
EPQ								
EPQ.P	-0.314***	-0.351***	-0.347***	-0.281***	-0.091**	-0.336***	-0.402***	-0.426***
EPQ.E	0.077**	0.125***	0.150***	0.187***	0.386***	0.223***	＜0.001	0.228***
EPQ.N	-0.330***	-0.178***	-0.102***	-0.362***	-0.153***	-0.197***	-0.248***	-0.319***
DSI-R								
ER	0.197***	0.016	-0.03	0.290***	0.114***	0.025	0.026	0.131***
IP	0.288***	0.279***	0.378***	0.431***	0.332***	0.358***	0.287***	0.472***
EC	0.288***	0.247***	0.241***	0.206***	0.157***	0.235***	0.303***	0.337***
FO	0.295***	0.051	-0.011	0.291***	0.132***	0.083**	0.146***	0.203***
DSI-R Total Score	0.407***	0.197***	0.173***	0.450***	0.258***	0.234***	0.272***	0.404***

Note: CTDI-CV: Critical Thinking Disposition Inventory-Chinese Version; EPQ: Eysenck Personality Questionnaire; P: Psychoticism; E: Extraversion; N: Neuroticism; DSI-R: Differentiation of Self-Revised; ER: Emotional Reactivity; IP: I-Position; EC: Emotional Cutoff ; FO: Fusion with Others. *$p < 0.05$. **$p < 0.01$. ***$p < 0.001$.
